# Multi-endpoint analysis of cadmium chloride-induced genotoxicity shows role for reactive oxygen species and p53 activation in DNA damage induction, cell cycle irregularities, and cell size aberrations

**DOI:** 10.1093/mutage/gead025

**Published:** 2023-08-09

**Authors:** Leanne M Stannard, Ann Doherty, Katherine E Chapman, Shareen H Doak, Gareth J Jenkins

**Affiliations:** In Vitro Toxicology Group, Institute of Life Science, Swansea University Medical School, Swansea University, Swansea SA28PP, United Kingdom; Safety Innovation, Astra Zeneca, Cambridge, United Kingdom; In Vitro Toxicology Group, Institute of Life Science, Swansea University Medical School, Swansea University, Swansea SA28PP, United Kingdom; In Vitro Toxicology Group, Institute of Life Science, Swansea University Medical School, Swansea University, Swansea SA28PP, United Kingdom; In Vitro Toxicology Group, Institute of Life Science, Swansea University Medical School, Swansea University, Swansea SA28PP, United Kingdom

**Keywords:** DNA damage, genotoxicity

## Abstract

Cadmium chloride (CdCl_2_) is a known genotoxic carcinogen, with a mechanism of action thought to partly involve the generation of reactive oxygen species (ROS). We applied here a multi-endpoint approach *in vitro* to explore the impact of CdCl_2_ on both the genome and on wider cell biology pathways relevant to cancer. Multi-endpoint approaches are believed to offer greater promise in terms of understanding the holistic effects of carcinogens *in vitro*. This richer understanding may help better classification of carcinogens as well as allowing detailed mechanisms of action to be identified. We found that CdCl_2_ caused DNA damage [micronuclei (MN)] in both TK6 and NH32 cells in a dose-dependent manner after 4 h exposure (plus 23 h recovery), with lowest observable effect levels (LOELs) for MN induction of 1 μM (TK6) and 1.6 μM (NH32). This DNA damage induction in TK6 cells was ROS dependent as pretreatment with the antioxidant *N*-Acetyl Cysteine (1 mM), abrogated this effect. However, 2ʹ,7ʹ-dichlorofluorescin diacetate was not capable of detecting the ROS induced by CdCl_2_. The use of NH32 cells allowed an investigation of the role of p53 as they are a p53 null cell line derived from TK6. NH32 showed a 10-fold increase in MN in untreated cells and a similar dose-dependent effect after CdCl_2_ treatment. In TK6 cells, CdCl_2_ also caused activation of p53 (accumulation of total and phosphorylated p53), imposition of cell cycle checkpoints (G2/M) and intriguingly the production of smaller and more eccentric (elongated) cells. Overall, this multi-endpoint study suggests a carcinogenic mechanism of CdCl_2_ involving ROS generation, oxidative DNA damage and p53 activation, leading to cell cycle abnormalities and impacts of cell size and shape. This study shows how the integration of multiple cell biology endpoints studied in parallel *in vitro* can help mechanistic understanding of how carcinogens disrupt normal cell biology.

## Introduction

Cadmium (Cd) is a metallic element which occurs naturally in the Earth’s crust. It is a toxic heavy metal with many industrial and commercial uses, leading to widespread environmental and occupational pollution [1]. Exposure to Cd occurs mainly through occupational and environmental exposure, as well as through food and tobacco smoke [2] it has been linked to cancer, as well as respiratory and neurological problems. Based on previous human and animal data, the International Agency for Research on Cancer(IARC) have classified Cd (including CdCl_2_) as a Group 1 human carcinogen [3].

In ocean water, Cd occurs at levels of <5 to 110 ng/l [4]. It is emitted to the environment as a result of both natural and anthropogenic activities, such as volcanic activity, weathering of Cd-containing rocks, sea spray, mining, and fossil fuel combustion [4, 5]. As the toxic effects of Cd became apparent, industrial limits on Cd exposure were imposed and it was replaced in many industrial activities, emissions of Cd have reportedly decreased by about half in Europe and two-thirds in Canada [5].

Acute exposure can cause flu-like symptoms, also known as the ‘cadmium-blues’, which usually pass in a week. More severe exposures, however, can cause respiratory damage such as bronchitis and pneumonitis, and direct ingestion of Cd can cause fatal respiratory tract and kidney problems, such as renal failure. Exposure to Cd can occur in an occupational setting, usually via the respiratory tract [6]. Cd metal has many specific properties which make it suitable for a range of industrial applications [7]. These include corrosion resistance, low melting temperature, high ductility, high thermal, and electrical conductivity [3]. Cd is widely used in industrial applications such as in the aerospace industry, automotive systems and military equipment and in Ni–Cd batteries. CdCl_2_ is used for the preparation of cadmium sulphide [8], and is also widely used in photocopying, dyeing, photography, and fabric printing.

Cd compounds have shown carcinogenic potential in both animals and humans. All Cd compounds, including CdCl_2_, have been classified as genotoxic carcinogens [3]. The IARC classification for Cd and Cd compounds, including CdCl_2_, as a Group 1 human carcinogen was based on animal and human studies, where there was significant evidence of increased lung cancer risk among workers occupationally exposed to Cd [3].

Cd-exposed workers from 17 plants in the UK were studied by Kazantzis *et al*. who noted a significant increase in mortality from lung cancer [9]. They also found a correlation between duration of employment and intensity of exposure with the increasing death rates [10]. studied Ni–Cd battery workers and confirmed an increase in mortality rates from lung cancer, which was also thought to be associated with duration of employment [10, 11]. Similar results were seen in studies by Elinder *et al*. and Jarup *et al.* who studied the effect a cumulative exposure to Cd had on lung cancer incidence, and found a dose-related response [12, 13]. The evaluation of the relationship between occupational exposure to Cd and cancer risk is inadequate due to a limited number of long-term, highly exposed workers and the lack of information on smoking status or co-exposure of the workers to other potential toxins/carcinogens [7].

Similar positive results have been observed in animals, with the studies performed providing sufficient evidence of carcinogenicity in animals showing that Cd compounds induce tumours by multiple routes, at various exposure concentrations, and at various sites in different species of experimental animals [7]. CdCl_2_ has been widely studied in animals, with oral administration to rats increasing the incidence of leukaemia, prostate, and testis tumours [14]. As well as oral exposure, CdCl_2_ has been shown to cause lung tumours in rats in inhalation studies, and injection-site sarcomas, lymphomas, and lung tumours in subcutaneous administration studies in rats and mice [15].

Although the mechanism of Cd-induced toxicity/carcinogenicity is not fully understood, Cd is known to cause genome instability [4, 16]. This instability can arise through multiple mechanisms including DNA repair inhibition (interference with zinc finger proteins) [17], ROS generation [18], mitochondrial toxicity [19], and direct reactivity with DNA phosphate groups [20]. Cd causes significant DNA strand breaks and chromosomal aberrations *in vitro* in mammalian cells, and is thought to be weakly mutagenic *in vitro* [3]. There are many studies which confirm the genotoxic potential of CdCl_2_ in mammalian cells *in vitro* [21]. In contrast, when tested in bacterial assays, Cd compounds are not mutagenic, and produce mainly negative responses, showing that Cd’s toxicity may be mammalian cell specific [3]. The genotoxicity of Cd salts is thought to arise via more indirect mechanisms, based on evidence that Cd salts do not cause DNA damage in cell extracts or with isolated DNA [22]. Many Cd compounds have been shown to induce oxidative stress *in vivo* and *in vitro* [18, 23–25]. Studies have also shown that Cd can reduce antioxidant defence mechanisms, thus increasing the production of ROS indirectly [18, 24, 26].

Due to some remaining uncertainty regarding the mechanism of Cd’s genotoxicity, we deployed a multi-endpoint approach based on the Hanahan and Weinberg Hallmarks of cancer [27] to further investigate CdCl_2_’s mechanism of genotoxicity/carcinogenicity *in vitro*. We specifically studied low-dose Cd effects to remove any confounding issues related to toxicity. We studied DNA damage, cell signalling errors, cell cycle irregularities, oxidative stress, and cell morphology perturbations, induced by Cd as previously described [28].

## Materials and methods

We employed the human lymphoblastoid cell line TK6, a derivative of the WIL-2 cell line. The cells are heterozygous at the TK locus and contain the wild-type TP53 gene. TK6 cells were acquired from the ECACC, Salisbury, UK (Cat.-No. 95111735). TK6 cells were kept between passages 10–15 throughout the experiments. The NH32 cell line was received as a gift to our laboratory from Prof. Dr. Gerald N. Wogan (MIT, Cambridge, MA, USA). Like TK6, the human lymphoblastoid cell line NH32 is a derivative of the WIL-2 cell line. The cells contain a double p53 knockout.

Both cell lines were cultured in RPMI 1640 (Gibco, UK) supplemented with 10% horse serum (Gibco, UK) and 1% l-Glutamine (Gibco, UK). All cell lines were maintained in a humidified incubator at 37°C with 5% CO_2_. The cells were maintained in exponentially growing cultures at a concentration of between 1–3 × 10^5^ cells/ml and did not exceed 1 × 10^6^ cells/ml. Upon reaching confluency, cells suspensions were counted with the Z1 Coulter Particle Counter (Beckman Coulter Inc., UK) and diluted with prewarmed growth media to a final concentration of approximately 1–3 × 10^5^ cells/ml.

All cell suspensions prior to chemical exposure were seeded at 1 × 10^5^ cells/ml into 25 cm^3^ flasks for 24 h at 37°C and 5% CO_2_. Each flask was dosed with appropriately diluted test chemical and placed into the incubator for the correct time point. After the exposure, the suspensions were transferred to labelled centrifuge tubes and spun for 10 min at 800 rpm and washed twice with prewarmed phosphate-buffered saline (PBS) to remove any residual chemical. Cells were resuspended in 10 ml of growth media and transferred to 25 cm^3^ flasks, cytochlasin B (final concentration 6 μg/ml) was added to the test flasks for one cell cycle.

### Test chemical

CdCl_2_ was acquired from Sigma-Aldrich, UK. The chemical was freshly diluted with water before each use.

### The *in vitro* micronuclei assay

After treatment with CdCl_2_ and incubation with Cytochalasin B for one cell cycle, the contents of the tissue culture flasks were transferred into 15 ml centrifuge tubes and washed with PBS. The cells were then treated with 0.56% KCl solution (hypotonic), centrifuged immediately for 10 min at 800 rpm at 4°C and the supernatant was discarded. Cells were then fixed with methanol/acetic acid/0.09% NaCl (5:1:6) solution for 10 min, centrifuged and the supernatant discarded. Next, a second fixation step was performed by treating the cells with methanol/acetic acid (5:1) for 10 min, after which cells were centrifuged and the supernatant discarded. This second fixation wash step was repeated four times, and cells were incubated in fixative 2 for 16 h at 4°C. The fixed cells were dropped against the length of labelled slides, and prepared for analysis by mounting in vectashield anti-fading solution containing 4ʹ,6-diamidino-2-phenylindole (DAPI) stain, and covered with a cover-slip (25 × 60 mm, VWR International). The slides were scanned at 10× magnification with the Metafer 4 master station, coupled to an Olympus BX50 fluorescent microscope (Carl Zeiss) as previously described [28]. Samples were prepared in triplicate, with 3000 cells scored per replicate (9000 cells per dose).

### Cytotoxicity

Cell viability of TK6 cells was determined using relative population doubling (RPD) measurements. Parallel cell cultures exposed to CdCl_2_ were used to generate the RPD data.

RPD was calculated as follows:


$$\begin{array}{l} RPD\;=\;\frac{Number\;of\;population\;doublings\;in\;treated\;cultures}{Number\;of\;population\;doublings\;in\;control\;cultures}\;\times \;100 \end{array}$$

$$\begin{array}{l} Population\;doubling\;=\;\frac{\;[\;log(posttreatment\;cell\;count/initial\;cell\;count)\;]\;}{log\;2} \end{array}$$

### Western blotting

Posttreatment with CdCl_2_, the cell suspensions were transferred to labelled centrifuge tubes at spun at 800 rpm for 10 min. The supernatant was discarded and the cells were washed twice in ice-cold PBS. After resuspension of the pellet, 200 μl of radioimmuno-precipitation lysis (RIPA) buffer (Sigma-Aldrich, UK) supplemented with 2 μl of phosphatase inhibitor cocktail (Sigma-Aldrich, UK) were added and the cell suspensions were transferred to prechilled micro-centrifuge tubes and incubated for 5 min at 4°C. After this incubation, the celled were lysed by vortexing thoroughly and centrifuged for 10 min at 10 000 × *g* in a precooled (4°C) centrifuge.

The Pierce Assay was used for the protein quantification using the Pierce^TM^ BCA Protein Assay Kit (Thermo Scientific, Life Technologies, UK). Protein samples were mixed in a 1:1 ratio with 4°C Laemmli buffer (Sigma-Aldrich, UK), sonicated 3 × 10 s with 10 s intervals, and then incubated for 5 min at 95°C. The protein samples were then loaded into the wells of the 10% gel (with 4% stacking gel), as well as 8 μl of the Dual Colour Standard and Biotinylated Ladder. Following transfer onto polyvinylidene difluoride (PVDF) membrane (BioRad), the membranes were carefully removed from the transfer cassette, washed in Tris-buffered saline / Tween (TBS/T) wash buffer, and incubated in blocking buffer (5% Bovine serum albumin BSA) for 1 h at room temperature. The membranes were then incubated with primary antibody, diluted 1:1000 in 5% BSA blocking buffer overnight at 4°C with gentle agitation. Finally, the membranes were washed with TBS/T for 4 × 5 min at room temperature with agitation, and then incubated for 1 h in HRP conjugated goat polyclonal secondary antibody to rabbit Immunoglobulin G(IgG) (1:1000 dilution; Abcam, UK) and Antibiotin horseradish peroxidase(HRP) linked antibody (1:1000 dilution; Cell Signalling, UK) with gentle agitation at room temperature.

Proteins were detected using the Immun-Star Western C Chemiluminescent kit (BioRad, UK). Finally, band densitometry was determined using a ChemiDoc (BioRad) and QuantityOne software (BioRad, UK) and the sample band densities were normalized against the bands for the corresponding house-keeping gene, Beta-actin.

### Real-time PCR

Total RNA was extracted from the human lymphoblastoid cell lines using the Qiagen RNeasy mini kit (Qiagen, UK), following manufacturers instructions. The concentration of RNA was then measured, and the purity was assessed (260:280 ratio) using the NanoDrop ND-1000 Spectrophotometer (Labtech International, UK).

To convert RNA to cDNA, the Quantitect Reverse Transcription kit (Qiagen) was used. A gDNA elimination reaction was performed as a single reaction The Quantifast SYBR Green Kit (Qiagen) was used to perform quantitative real-time Polymerase Chain Reaction (qPCR). Mastermixes containing the SYBR Green PCR Master Mix, primers from [28] and H_2_O were prepared for the above reactions to ensure equal distribution of components between wells. All reactions were performed in triplicate. Standard samples and a negative control, where cDNA was replaced with H_2_O, were included on every plate. The levels of the house-keeping gene, B-actin, were also analysed. After the plate was loaded, it was sealed with adhesive film and centrifuged at 1000 rpm for 1 min to settle the contents. The iCycle IQ5 thermal cycler (BioRad) was then used.

### Cell cycle analysis

The cell cycle status, in cells treated with CdCl_2_, was analysed by quantitation of DNA content using the Litron MicroFlow kit (Litron Laboratories) and flow cytometry. All cell suspensions (10 ml) were seeded at 1 × 10^5^ cells/ml for 24 h at 37°C, 5%CO_2_ and treated with test chemicals. The treated cells were spun down (10 min, 800 rpm), washed with PBS to remove any residual chemical and resuspended in PBS, before determining the cell concentration using the Beckman Coulter Counter (Beckman Coulter Inc., UK). Then, 1 × 10^6^ cells were transferred to new tubes ready for staining with the cell cycle stain from the Litron MicroFlow kit as per the manufacturer’s instructions. Samples were analysed using the BD FACS Aria Flow Cytometer (BD Biosciences), with FacsDiva software (BD Biosciences), within 72 h of harvesting. Appropriate gating was applied to determine the live cell population. A total of 30 000 events were analysed across three replicates per dose.

### High content analysis of cell size/shape

Posttreatment with CdCl_2_, the cell suspensions were transferred to labelled centrifuge tubes at spun at 800 rpm for 10 min. Samples were then resuspended in 10 ml PBS and this washing process repeated. To undergo cell fixation, the cells resuspended in 200 μl of 4% paraformaldehyde (diluted in PBS) for 15 min at room temperature. Following this, 3 ml PBS was added to each tube, samples were centrifuged and the supernatant aspirated. This washing process with 3 ml PBS was repeated once again. Samples were then resuspended in 3 ml PBS and stored at 4°C until staining with Hoechst 33342, to a concentration of 2.5 µg/ml. To prevent agglomeration, pellets were then mixed thoroughly by slowly pipetting the samples, and were then incubated for 30 min at room temperature while protected from light. Following this, cells were washed twice in 3 ml PBS, and resuspended in 1–5 ml PBS, depending on the pellet size. The concentration of cells was then recorded using a Z1 Coulter Counter.

Cell samples were aliquoted into a 24-well CellStar plate, ensuring a total concentration of 100 000 cells/ml with a total volume of 1.1 ml. Analysis with the InCell Analyzer 2000 was carried out within 2 days. A total of 144 images were collected for each well, with the total magnification set to 200×. A second image was acquired for each field-of-view using the DAPI excitation and emission filter set to identify nuclei (Hoechst stain).

Images were analysed using a Matlab script, which recorded details into cell and nuclear area, perimeter, solidity, eccentricity, and form factor (as previously reported—[28]). An equal number of cell and nuclear area results were selected from a group of control replicates. These control groups were segregated depending on experimental conditions, vehicle and cell type. The smallest 20% of the population were then classified as ‘Lowest’, the next 20% as ‘Low’ and so on to classify ‘Medium’, ‘High’, and ‘Highest’ cellular/nuclear area thresholds (quintiles).

### Reactive oxygen species

Reactive oxygen species (ROS) detection was undertaken using the fluorescent 2ʹ,7ʹ-dichlorofluorescin diacetate (DCFDA) assay (Sigma-Aldrich). Cells were seeded at 1 × 10^5^ and incubated for 24 h. On the day of treatment, the test chemical and positive control were diluted accordingly. The 4000× DCFDA stock was diluted to 1× DCFDA, and 100 µl was added to each well in a 96-well plate. The cell flasks were treated with CdCl_2_, and a 100 µl aliquot was immediately added to the 96-well plate. Fluorescence readings were taken using a FLUOstar Omega Multimode microplate reader (BMG LABTECH Ltd, UK) with excitation/emission set at 485/535 at various time points from the time of dosing; 10 min, 15 min, 30 min, 1 h, 2 h, 4 h, 6 h, and 24 h. Hydrogen peroxide (50 μM) was used as a positive control in all experiments.

### Statistical analysis

A Levene’s test was firstly carried out to confirm an equal variance between samples. Then a one-way analysis of variance with Dunnett’s *post hoc* test, or a Kruskal–Wallis and Dunn’s test, was used to assess whether statistically significant (*P* < 0.05) differences existed between vehicle control and treated samples in many of the different endpoints.

All error bars represent standard deviation around the mean of the biological replicates.

## Results

### Cadmium chloride-induced genotoxicity in TK6 and NH32 cells *in vitro* as measured by the micronucleus assay

**Figure 1.**
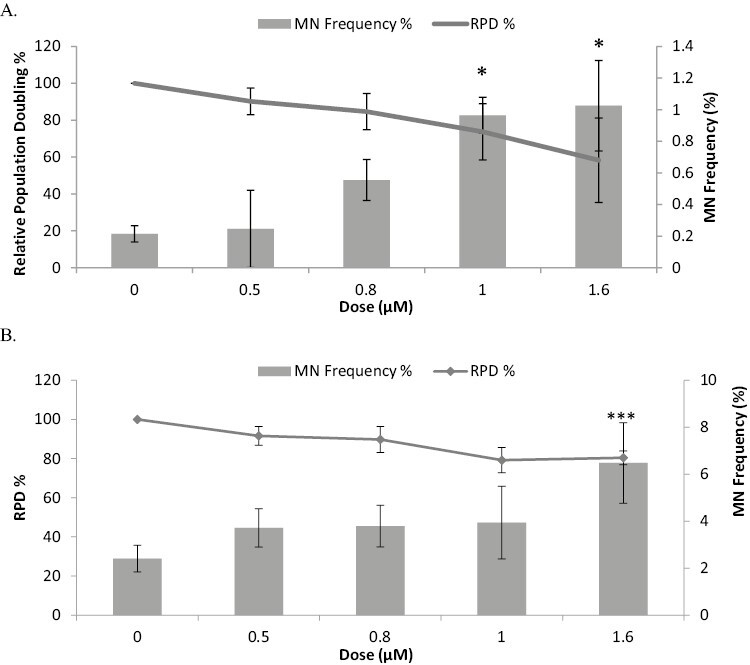
Cytokinesis blocked Micronucleus Assay in TK6 cells (A) and NH32 cells (B) after exposure to CdCl_2_ for 4 h (plus 23 h recovery). LOEL for Mn induction 1 μM (TK6) and 1.6 μM (NH32). Error bars represent standard deviation, *n* = 3. Significance analysed using Dunnett’s t-test: *P ≤ 0.05, **P ≤ 0.01, and ***P ≤ 0.001.

To explore the DNA-damaging potential of CdCl_2_, human lymphoblastoid TK6 cells were treated with CdCl_2_ and the *in vitro* Cytokinesis blocked Micronucleus Assay (CBMN) assay was performed to investigate chromosomal damage. As stated in the OECD guideline 487 [29] the test concentrations selected for the micronuclei (MN) assay should cover a range of producing <50 ± 5% toxicity to little or no toxicity, hence we deliberately focussed on the low dose range of CdCl_2_ (<2 µM). Chromosome damage induction was measured in TK6 cells using the CBMN assay (Fig. 1A), with cytotoxicity measured in parallel via RPD calculations, a sensitive measure of cytotoxicity. Cells were treated with CdCl_2_ for 4 h (+23 h or 1.5 cell cycles, for recovery) to generate a dose response and identify the lowest observable effect level (LOEL).

CdCl_2_ caused a dose-dependent decrease in cell viability, with 58% RPD reached at 1.6 µM (Fig. 1A). Figure 1 also shows a dose-dependent increase in genotoxicity, with the genotoxic LOEL observed at 1 µM CdCl_2_, this being a 4.5-fold increase in MN level above the control. A similar response profile was seen when using the isogenic, p53 deficient NH32 cell line (Fig. 1B). Cell viability was less affected in NH32 cells, with CdCl_2_ causing half as much toxicity in NH32 cells compared with TK6 cells (viability 80% in NH32 cells at top dose compared with 58% in TK6). The LOEL for MN (1.6 µM) determined in NH32 cells was higher than in the TK6 cell line (1 µM), however, the background %MN was far higher in these p53 deficient cells (>10-fold higher than TK6 cells 2% vs. 0.2%). There was a statistically significant difference in sensitivity to MN induction between the two cell lines tested (*P* ≤ 0.001). The highest CdCl_2_ dose (1.6 µM) induced over 6% Mn in NH32 cells, compared with a Mn induction in TK6 cells of only 1% at this same dose. These observations demonstrate overt genome instability in the p53-deficient NH32 cells.

### Cadmium chloride does not induce measurable levels of reactive oxygen species, but antioxidant supplementation reversed DNA damage induction

**Figure 2.**
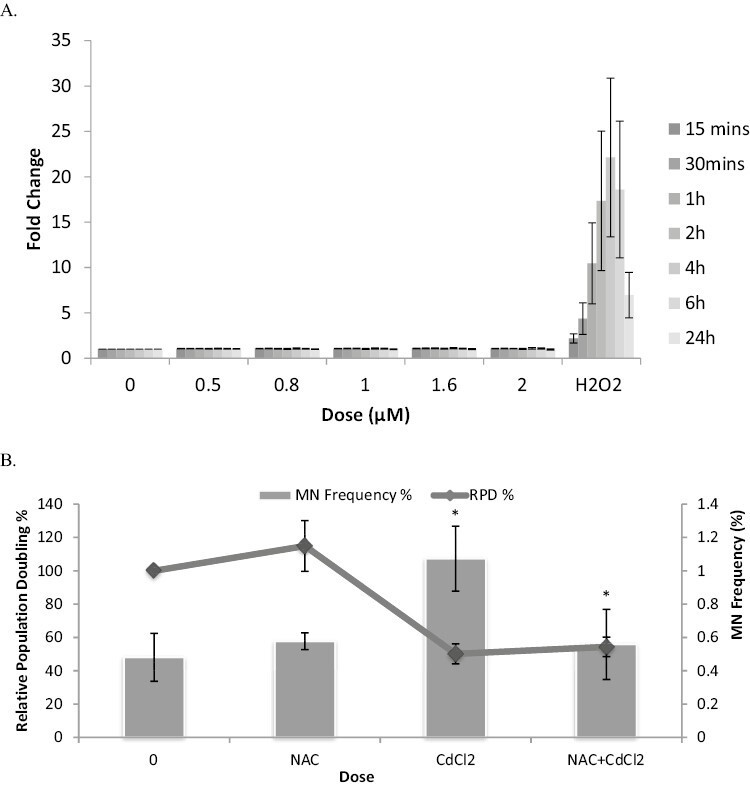
(A) DCFDA analysis of CdCl_2_ induced ROS. No ROS induction was observed across this dose range up to 24 h postexposure. Error bars represent standard error, *n* = 3. Significance analysed using Dunnett’s t-test: *P ≤ 0.05, **P ≤ 0.01 ***P ≤ 0.001. (B) Micronucleus induction by CdCl_2_ with and without 24 h pretreatment with *N* Acetyl Cysteine (1 mM) showing abrogation of CdCl_2_ induced MN, suggestive of oxidative DNA damage involvement. Significance analysed using Dunnett’s t-test: *P ≤ 0.05, **P ≤ 0.01, and ***P ≤ 0.001.

As ROS induction has been previously suggested to mediate CdCl_2-_induced DNA damage, this was explored further through two approaches with TK6 cells. Firstly, the levels of ROS induced by treatment with low doses of CdCl_2_ was analysed via the addition of the cell-permeable ROS sensor DCFDA (Fig. 2A). After an acute treatment of low doses of CdCl_2_, for 4 h at doses up to 2 µM, no significant ROS induction was observed at any of the doses tested up to 24 h postexposure. A highly significant response was seen with the positive control, hydrogen peroxide.

Secondly, we used an antioxidant approach to assess if CdCl_2-_induced Mn were abrogated by pretreatment with the antioxidant *N*-Acetyl Cysteine (NAC), which would be suggestive of an oxidative DNA damage origin. A 4 h treatment (+23 h recovery) with CdCl_2_ induced a significant increase in MN frequency (Fig. 2B). We also observed that following a NAC pretreatment of 1 mM for 24 h, the MN induction seen after treatment with CdCl_2_ was significantly decreased back to the background level (Fig. 2B). CdCl_2_ treatment alone produced a MN frequency of 1.1% which, after a pretreatment, was reduced to 0.55% (background = 0.48%). Interestingly, the NAC supplementation did not reverse the CdCl_2_-induced toxicity (reduction in %RPD), which may highlight a nonROS-mediated cytotoxic effect.

### Cadmium chloride caused significant activation of p53 and p-p53 levels

**Figure 3.**
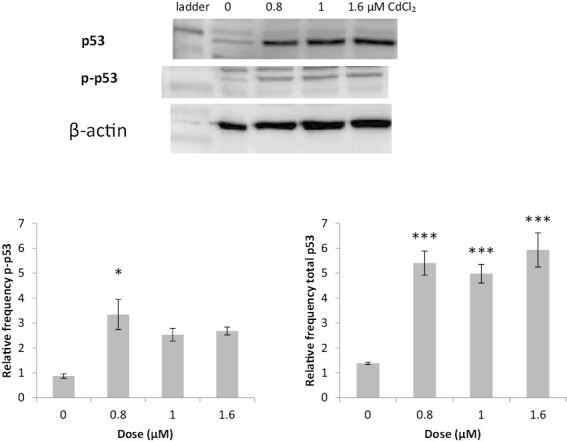
Illustrative Western blot showing P53 stabilization by CdCl_2_ (top panel), Beta Actin used as the housekeeper. Graphs (TK6 cells) showing a dose-dependent increase in both phospho-p53 (Ser15) (left-hand graph) and total p53 protein (right-hand graph) after exposure to CdCl_2_ (triplicate data). Error bars represent standard deviation, *n* = 3. Significance analysed using Dunnett’s t-test: *P ≤ 0.05, **P ≤ 0.01, and ***P ≤ 0.001.

Both total and phosphorylated p53 (Ser15) levels in TK6 cells were analysed via western blotting after a 4 h treatment with CdCl_2_. Example Western blots are displayed in Fig. 3. Normalization of these protein expression values was carried out using the B-actin house-keeping gene, and the average calculated from three replicates (Fig. 3B). A statistically significant increase was seen in the expression of p53 protein levels after a 4 h treatment with CdCl_2_ at all doses tested (0.8, 1, and 1.6 µM). There was also a significant increase in p-p53 levels, although this was only observed at the lowest dose 0.8 µM. Interestingly, p53 stabilization occurs at doses of CdCl_2_ below the LOEL for Mn induction (Fig. 1A) suggesting that cell signalling alterations can be observed at lower doses than are detected using the MN assay.

Further analysis of cell signalling alterations induced by low-dose CdCl_2_ were performed by qPCR using a panel of genes previously identified by our group [28]. TK6 cells were treated for 4 h, after which RNA was extracted immediately for RT-PCR analysis. After a 4 h treatment with CdCl_2_, an increase was seen in the expression of p21 with fold changes of 1.3- and 1.5-fold, however, these did not reach statistical significance. No significant increases were seen in the expression of either SGK1 (Serum/Glucocorticoid Regulated Kinase 1) or CHKA (Choline Kinase Alpha) after treatment with CdCl_2_ at any doses tested (see Supplementary Fig. 1).

### Cadmium chloride caused significant changes in cell cycle, which were ROS dependent

**Figure 4.**
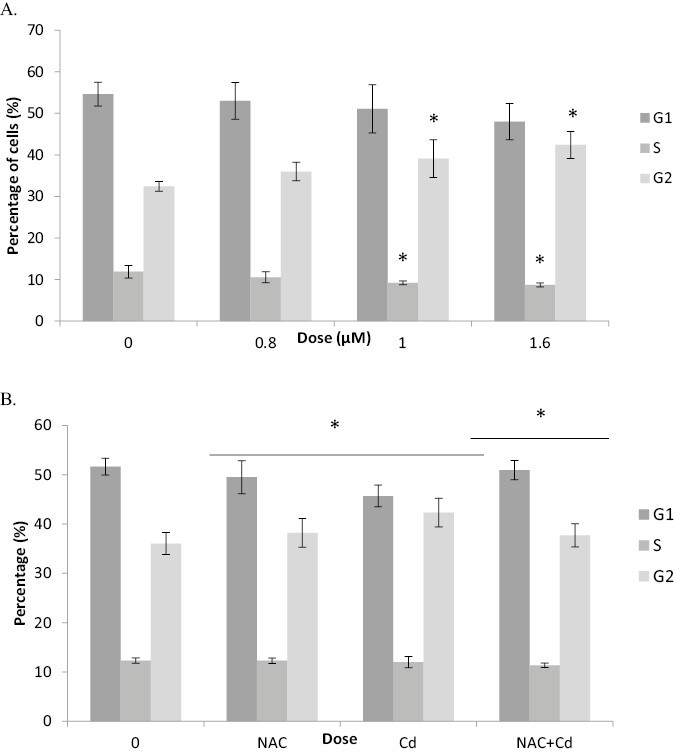
CdCl_2_ exposure (1.6 µM treatment for 4 h + 23 h) induced significant changes to the cell cycle distribution. A nonsignificant decrease in cells in G1 phase, a decrease in S phase cells and an increase of cells in G2 phase were noted (A). Pretreatment with NAC reversed the increase in G2 cells induced by CdCl_2_ suggesting a ROS-mediated basis (B). Error bars represent standard deviation, *n* = 3. Significance analysed using Dunnett’s t-test: *P ≤ 0.05, **P ≤ 0.01, and ***P ≤ 0.001.

The proportion of TK6 cells in each phase of the cell cycle (G1, S, and G2/M) was determined after CdCl_2_ treatment, via a flow cytometric approach using the Litron Microflow reagents. This data was collected using a treatment time of 4 h and a recovery time of 23 h (1.5 cell cycles), to give the cells enough time to progress through a full cell cycle. As shown in Fig. 4A, after treatment with CdCl_2_ the % of TK6 cells in S phase of the cell cycle decreased in a dose-dependent manner from 11.9% (control) to 8.7% (top dose of 1.6 μM). This decrease reached statistical significance at 1 and 1.6 μM CdCl_2_. This decrease may be explained by a parallel dose-dependent increase in 4N cells in the G2/M phase; increasing from 32.4% (control) to 39.1% and 42.4% at 1 and 1.6 μM CdCl_2_.

As noted above with the Mn data (Fig. 2B), pretreatment with 1 mM NAC reversed the CdCl_2_-induced cell cycle changes (Fig. 4B). After treatment with CdCl_2_, the % of cells increased from 36% in the control to 42.3%, however, after a pretreatment with 1 mM NAC this was reduced back down to 37.7% (Fig. 4B). These results suggest that CdCl_2_ induced a G2/M block which was ROS dependent.

### Cadmium chloride caused significant cellular and nuclear morphological alterations

**Figure 5.**
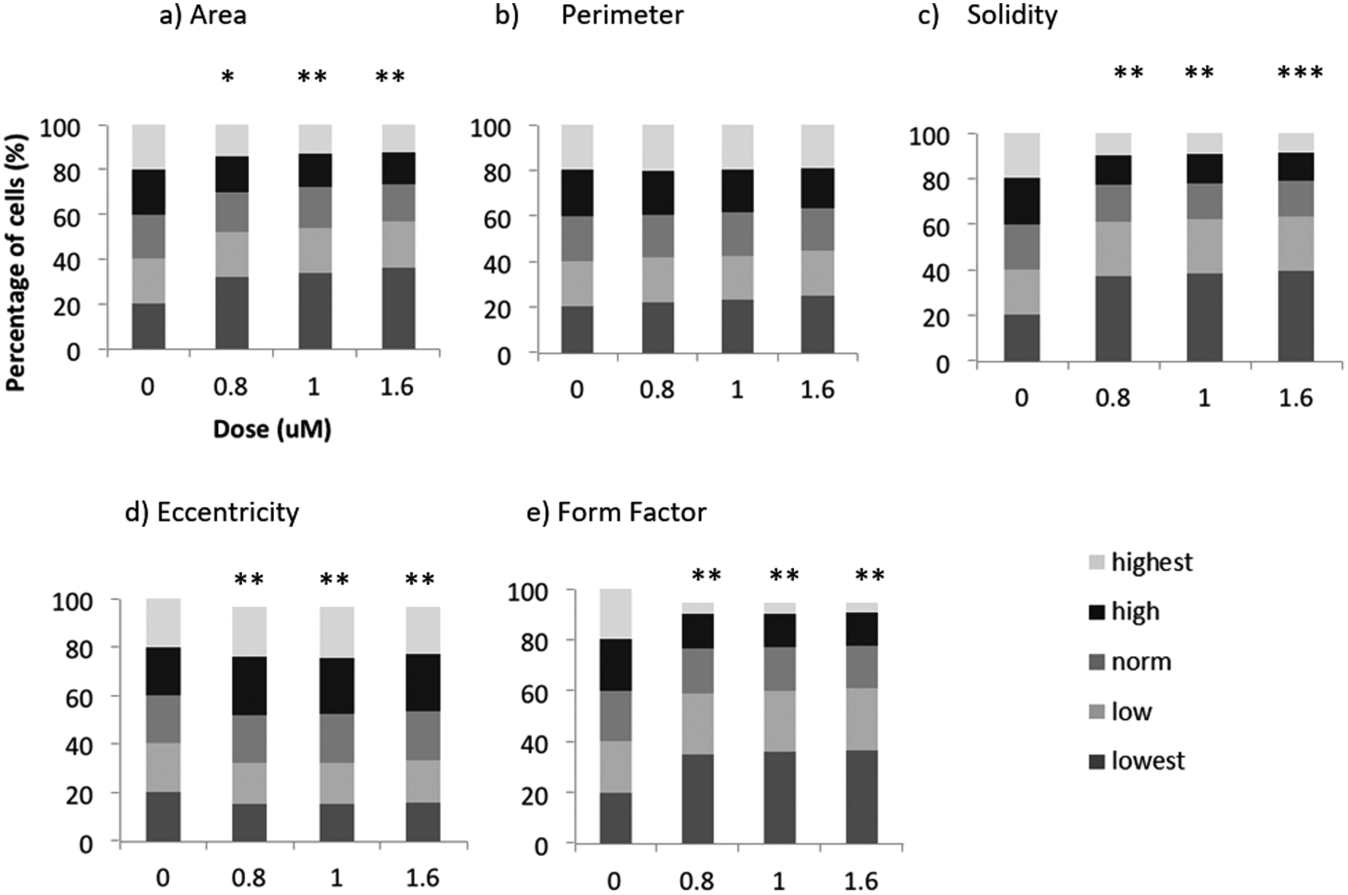
Cellular alterations following a 4 h treatment (no recovery time) with CdCl_2_. (a) cell area, (b) cell perimeter, (c) cell solidity, (d) cell eccentricity (elongation), and (e) cell form factor (circularity). Images acquired using InCell Analyzer 2000 (*n* = 3) and analysed with Matlab. Significance was assessed using Dunnett’s t-test or Dunn’s test: *P ≤ 0.05, **P ≤ 0.01, and ***P ≤ 0.001.

In order to assess global changes to cell morphology and behaviour, a high content analysis (HCA) of cellular and nuclear size and shape was undertaken after exposure of TK6 cells to CdCl_2_. After treatment with CdCl_2_, significant changes were noted in cellular area, solidity, eccentricity and form factor (Fig. 5). These cellular effects were evident at all doses suggesting that cell size was a sensitive endpoint for Cd-induced effects. We observed a global effect of Cd on cell size and shape, with cells tending to be smaller and more elongated after Cd treatment compared with untreated cells.

Unlike the changes observed in cellular morphology, no significant alterations were seen in nuclear area, perimeter, form factor or solidity (Supplementary Fig. S2). There was, however, a significant change in the nuclear eccentricity, which relates to the level of elongation shown in the nuclear outline. There was a significant reduction in the number of cells in the ‘lowest’ category after a treatment with 0.2 and 0.3 μM of CdCl_2_. This indicates that nuclear eccentricity was increased after treatment with CdCl_2_, with the same result being observed in cellular eccentricity (Fig. 5).

## Discussion

The aim of this investigation was to apply a novel multi-endpoint *in vitro* approach to holistically assess the mechanisms underlying the carcinogenicity of low-dose CdCl_2_. Firstly, the genotoxic potential of CdCl_2_ was confirmed via the *in vitro* cytokinesis block MN assay in TK6 cells, which was then coupled to a number of key carcinogenicity-associated endpoints. This preliminary genotoxicity (and cytotoxicity) data was required to select concentrations for use with other endpoints (genotoxic anchoring). A significant MN induction was noted after a 4 h treatment with CdCl_2_ (+23 h recovery). Doses either side of the LOEL were calculated and used for the further studies, which included cell signalling, cell cycle, cell morphology and production of ROS.

### DNA damaging potential of cadmium chloride via CBMN assay

CdCl_2_ is described as a genotoxic carcinogen, based on previous studies. For example, CdCl_2-_induced DNA damage has recently been noted in HepG2 cells measured by both the Comet assay and the production of gamma H2AX [18, 30]. The positive MN induction observed here in the 4 + 23 h CBMN assay agree with this classification. The positive response seen here after a short exposure (4 h) to low-dose CdCl_2_ indicates that CdCl_2_ is a potent genotoxin.

The mechanism of CdCl_2_-induced genotoxicity has been suggested to be via the production of ROS [18, 23, 24, 31–33]. For example, in studies by [34], CdCl_2_ was shown to produce 8-oxodG adducts in the DNA of human lymphoblastoid cells [34]. Oxidative stress induced by CdCl_2_ has been noted in Chinese hamster ovary(CHO) cells, Hela and bovine endothelial cells postexposure [35, 36]. Studies also state that CdCl_2_ is indirectly genotoxic and induces oxidative stress from indirect processes, such as a decrease of cellular antioxidants [37]. A recent Toxtracker study has highlighted the ability of this system to detect CdCl_2_’s NF-kB activation (RTKN reporter), but not the DNA damage reporter (BSCL2) supporting an ROS mechanism driving NF-kB [38]. Here, however, a treatment with low-dose CdCl_2_ was not shown to induce a significant level of ROS as measured by the commonly used ROS sensor, DCFDA. Studies by Nair *et al.* describe the importance of the dose, duration and frequency of Cd exposure on the production of oxidative stress. They also explain how the presence or absence of serum in experimental conditions can alter the results, as well as the type of cells and their antioxidant capacity [39]. Cd is able to induce a variety of ROS including H_2_O_2_, O_2_^●–^ and ^●^OH. However, it may be that the dose used here was not high enough to induce a sufficient level of ROS, or that the 4h acute treatment was not a sufficient treatment time to enable detection with DCFDA. We deliberately used low doses of CdCl_2_ here to investigate low-dose effects, it may be the case that these doses were too low to measure induced ROS.

When the antioxidant defence in the human body becomes overwhelmed, oxidative stress is said to occur. The addition of antioxidants via a pretreatment is known to protect against, or reverse, detrimental oxidative effects caused by toxins/carcinogens. Here it was observed that after a 24 h pretreatment with the antioxidant NAC, a significant reduction was seen in the frequency of MN induction, as well as the abrogation of the Cd-induced G2/M block which was observed after treatment with CdCl_2_ alone. The protection against the damaging effects caused by CdCl_2_ corresponds to data in the literature, which states that CdCl_2_ acts via oxidative stress mechanisms [1, 18, 35–37, 39]. These results in the literature, as well as the results here, suggest that CdCl_2_ is indeed acting via ROS-dependent mechanisms. However, with no significant ROS induction noted, cell cycle aberrations involving G2/M and clear Mn formation, we cannot exclude an aneugenic mode of action.

### Cell signalling via real-time PCR and western blotting

The positive MN response shown after a 4 h treatment with CdCl_2_ was followed up with the study of cell signalling aberrations. The increase in levels of p53 (and phospho-p53) induced by CdCl_2_ may be explained by the chromosomal damage identified in the CBMN assay. Following treatment with CdCl_2_, a significant increase in total p53 and phospho-p53 levels was observed. It is unsurprising that the levels of these were increased as p53 has an anticancer function and its activation underlies a key cancer-protective signalling network which is triggered by DNA damage. To further study how p53 influences micronucleus induction, the response to CdCl_2_ treatment in NH32 cells was undertaken. These cells contain a double p53 knockout, and the increased background MN shown in Fig. 1B emphasizes the importance of p53 in DNA damage repair and genomic stability. There was a statistically significant increase in sensitivity to MN induction in NH32 compared with TK6 cells (*P* ≤ 0.001). Although the LOEL observed (1.6 μM) was higher in NH32 cells than in the TK6 cell line (1μM), the background MN frequency in the NH32 cell line was >10 times higher, as the cells are much less stable. NH32 cells are clearly sensitive to the DNA damage induced by CdCl_2_ (with >6% MN induced at highest dose) further emphasizing the role of p53 in Cd-induced genotoxicity. Interestingly, CdCl_2_ induced far less cytotoxicity in NH32 cells (as measured by RPD), this may well reflect the lack of CdCl_2-_induced apoptosis in this p53 deficient cell line (Fig. 1B). It has previously been noted that CdCl_2_ induces apoptosis in cells and has been seen to activate the effector caspases driving apoptosis [18, 40], hence this pro-apoptotic effect may be absent in the NH32 cells leading to reduced toxicity (cell viability).

Due to the significant increase in p53 and phospho-p53 levels observed by CdCl_2_ treatment, it is surprising that a corroborating increase in p21 levels was not observed in the qPCR study. Increased expression of the p21 cyclin-dependent kinase inhibitor was noted here, and elsewhere [41] but these were not significant, perhaps due to the low-dose exposures used, or perhaps the 4 h treatment is not sufficient to produce a clear p21 induction. Studies by Aimola *et al*. studying Cd-induced p53-dependent apoptosis, saw a significant dose-dependent increase in the levels of both p21 and p53 [42]. As well as these studies, the link between p53 activation and p21 is well documented and understood. For example, p53-mediated growth inhibition is known to be dependent on the induction of p21, an inhibitor of cyclin-dependent kinases that are required for cell cycle progression. In many cancers, mutant p53 fails to transactivate p21, which in turn leads to uncontrolled proliferation.

### Cell cycle arrest

DNA damage is known to trigger cell cycle checkpoints, to ensure that cells cannot progress to the next stage of the cell cycle when damage has occurred. Following a 4 h treatment with CdCl_2_ (+23 h recovery) a significant G2/M cell cycle block (and corresponding drop in cells in S phase) was observed. The DNA damage caused by CdCl_2_ identified in the CBMN assay and subsequent increases in p53 levels may explain the G2/M cell cycle block observed. The G2/M checkpoint ensures that DNA has been successfully replicated and is free of damage; cells that have damaged DNA are stalled in G2 and cannot recycle to S phase.

Present evidence suggests that CdCl_2_ induces this G2/M cell cycle block by directly stimulating p53 activity via ROS-dependent DNA damage response pathways. It has been shown in previous studies that many genes involved in cell cycle regulation are overexpressed following treatment with CdCl_2_ [36, 43].

Similar results were seen in studies by Yang *et al*. who studied the effect of Cd on cell cycle progression in CHO cells [44]. In their study, a 24 h treatment of 0.8 to 2 μM of CdCl_2_ caused a G2/M arrest similar to that described here. It would be interesting to understand the time dependence of our G2/M block as 23 h recovery was allowed before cell cycle analysis. At earlier time points posttreatment, more proximal G1/S phase blocks may also be observed.

### Cell morphological alterations

One of the earliest observations about cell size is its relationship to ploidy [45]. A higher proportion of cells in G2 phase of the cell cycle often correlates to a larger cell size, due to the increased amount of DNA content in the cells. Here, however, the G2/M block was not causing an increase in cell size. In contrast, when cell and nuclear morphological alterations were analysed after treatment with CdCl_2_ using a high-content analyser, a significant decrease in cell area and solidity was observed. This conflicting data could be due to cell population differences. Although 10% more cells (3000 of 30 000 cells analysed by flow cytometry) are seen in G2 after treatment with 1.6 μM of CdCl_2_, the HCA has determined that another 16% of cells are decreasing in size in parallel. Here, these 2 cell populations are being captured by two different systems (flow cytometry and InCell Analyzer) and are possibly being driven by 2 different mechanisms. The first mechanism is expected to be DNA damage causing a G2/M cell cycle block, which may be causing larger 4N cells. The mechanism causing a decrease in cellular size and area observed during the InCell analysis remains unknown. We have previously shown that nongenotoxic carcinogens can cause reductions in cell size [28]. It has been described by Fingar *et al*. that cell growth and cell cycle progression are coordinated but separable processes in mammalian cells [46]. It is also known that cell growth does not rely on cell cycle progression, and many previous studies have shown that when cell cycle events are blocked with chemicals or genetic lesions, cell growth continues unchecked [45–48]. Increasing the rate of cell cycle progression does not always accelerate mass accumulation, thus resulting in an abnormally small cell size [47–49].

As well as responses to DNA damage, the cell cycle also has surveillance mechanisms which monitor the size of the cells in each stage [50]. The existence of cell size checkpoints has been observed in G1 and G2, and it is, therefore, possible that the decrease in cell size seen after treatment with CdCl_2_ is causing the cells to be stopped in G2 phase of the cell cycle.

As well as changes in cell area and solidity, significant alterations were also noted in both cellular form factor and eccentricity. A similar change was also noted in nuclear eccentricity. Cellular form factor, or circularity, indicates a change in the ratio between area and perimeter. The decrease in cellular area, therefore, explains the decrease in circularity noted. Eccentricity relates to the elongation of a cell, with dividing cells known to have a larger elongation. After treatment with CdCl_2_, both nuclear and cellular eccentricity increases, indicating that the cells are elongated, this aspect would be a fertile area to follow up.

## Conclusions

We have investigated CdCl_2_, a genotoxic carcinogen, and further underlined the importance of a multi-endpoint approach to genotoxicity testing, with alterations occurring in numerous cancer-relevant endpoints tested. By combining the traditional genotoxicity data with other cell biological endpoints, we have allowed for a better mechanistic understanding of CdCl_2_ and highlighted the need for a more holistic approach to genotoxicity testing. The results discussed here suggest that CdCl_2_ is a genotoxic carcinogen via ROS-dependent mechanisms. The results also suggest that the ROS-mediated chromosomal damage caused by CdCl_2_ stimulated p53 expression, which in turn causes a G2/M cell cycle block and altered cell size and shape. It has also been shown that pretreatment with antioxidants can reduce effects, such as MN induction and cell cycle blocks caused by CdCl_2_, further improving our mechanistic understanding.

## Supplementary Material

gead025_suppl_Supplementary_Figures
