# Supplementary material for: Multi-endpoint analysis of cadmium chloride-induced genotoxicity shows role for reactive oxygen species and p53 activation in DNA damage induction, cell cycle irregularities, and cell size aberrations
Source: Mutagenesis. 2023 Aug 9;39(1):13–23. doi: 10.1093/mutage/gead025 (PMC10851103; doi:10.1093/mutage/gead025)
Supplement: gead025_suppl_Supplementary_Figures [file gead025_suppl_supplementary_figures.docx]

Figure S1. Gene expression data for selected gene targets show no changes in expression following treatment with CdCl_2_. Genes analysed taken from previous publication (Wilde et al., 2018). *Gene expression fold change following a 4h treatment with CdCl_2_. Gene expression was quantified using real-time PCR. Error bars represent standard deviation, n = 3. Significance analysed using Dunnett’s t-test: *P ≤ 0.05, **P ≤ 0.01 ***P ≤ 0.001.*

Nuclear Alterations
***
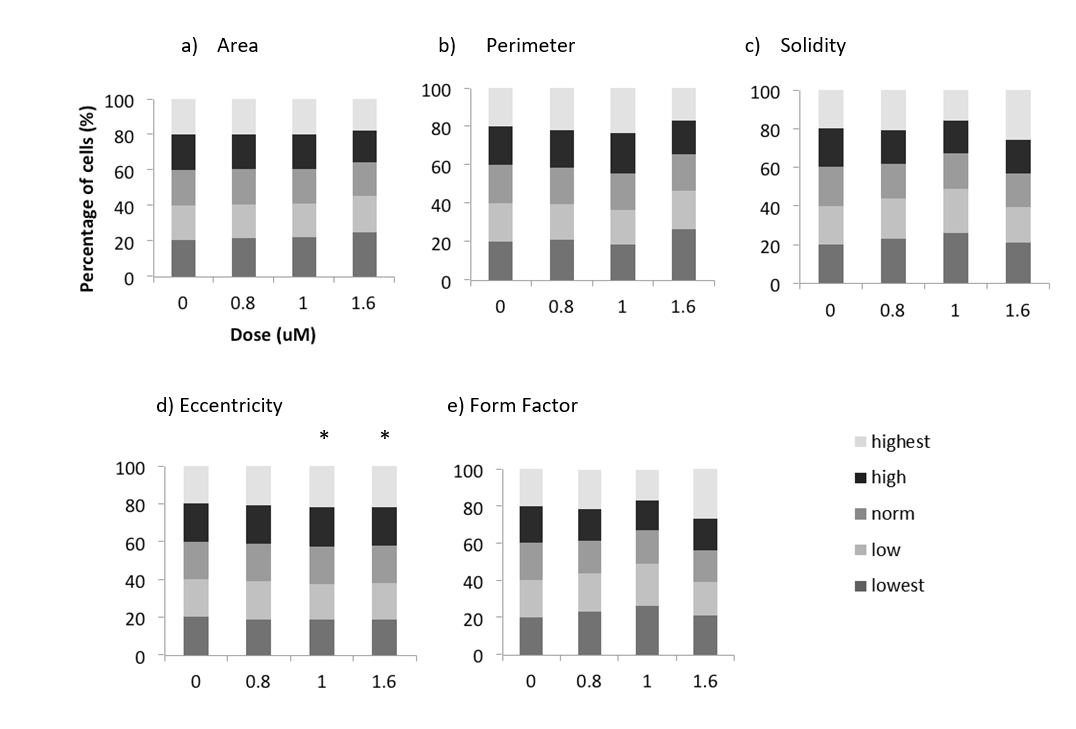
***

*Figure S2- Nuclear* *alterations* *following a 4 hour treatment (no recovery time) with CdCl_2_. (a) cell area, (b) cell perimeter, (c) cell solidity, (d) cell eccentricity and (e) cell form factor
Images acquired using InCell Analyzer 2000 (n = 3) and analysed with Matlab. Significance was assessed using Dunnett’s t-test or Dunn's test: *P ≤ 0.05, **P ≤ 0.01 ***P ≤ 0.001.*
